# Adaptive Method for Quantitative Estimation of Glucose and Fructose Concentrations in Aqueous Solutions Based on Infrared Nanoantenna Optics

**DOI:** 10.3390/s19143053

**Published:** 2019-07-11

**Authors:** Benjamin Schuler, Lucca Kühner, Mario Hentschel, Harald Giessen, Cristina Tarín

**Affiliations:** 1 Institute for System Dynamics and Research Center SCoPE, University of Stuttgart, Waldburgstr. 17/19, 70563 Stuttgart, Germany; tarin@isys.uni-stuttgart.de; 2 4th Physics Institute and Research Center SCoPE, University of Stuttgart, Pfaffenwaldring 57, 70569 Stuttgart, Germany; Lucca.Kuehner@physik.uni-muenchen.de (L.K.); m.hentschel@pi4.uni-stuttgart.de (M.H.); giessen@physik.uni-stuttgart.de (H.G.)

**Keywords:** glucose, fructose, glucose sensor, bio sensing, surface-enhanced infrared absorption, asymmetric least squares smoothing, superposition method

## Abstract

In life science and health research one observes a continuous need for new concepts and methods to detect and quantify the presence and concentration of certain biomolecules—preferably even in vivo or aqueous solutions. One prominent example, among many others, is the blood glucose level, which is highly important in the treatment of, e.g., diabetes mellitus. Detecting and, in particular, quantifying the amount of such molecular species in a complex sensing environment, such as human body fluids, constitutes a significant challenge. Surface-enhanced infrared absorption (SEIRA) spectroscopy has proven to be uniquely able to differentiate even very similar molecular species in very small concentrations. We are thus employing SEIRA to gather the vibrational response of aqueous glucose and fructose solutions in the mid-infrared spectral range with varying concentration levels down to 10 g/l. In contrast to previous work, we further demonstrate that it is possible to not only extract the presence of the analyte molecules but to determine the quantitative concentrations in a reliable and automated way. For this, a baseline correction method is applied to pre-process the measurement data in order to extract the characteristic vibrational information. Afterwards, a set of basis functions is fitted to capture the characteristic features of the two examined monosaccharides and a potential contribution of the solvent itself. The reconstruction of the actual concentration levels is then performed by superposition of the different basis functions to approximate the measured data. This software-based enhancement of the employed optical sensors leads to an accurate quantitative estimate of glucose and fructose concentrations in aqueous solutions.

## 1. Introduction

The reliable detection and identification of minute analyte concentrations in complex environments has been a long-standing problem, in particular in health care, life science, and point of care applications. In recent years the detection of glucose has attracted particular interest, as it is a highly important molecule in the human diet and metabolism. The, ideally, continuous monitoring of blood sugar levels is crucial for the treatment of, e.g., diabetes mellitus, with an ever growing number of patients [1,2,3,4]. This sensing task is, in fact, a very challenging one. Not only are the concentrations of glucose in the human blood or in the ocular fluid (as an easy-to-access system) very small, their detection is also hurdled by the extremely large number of additional molecular species in these body fluids [5,6,7]. A particular challenge lies with the other monosaccharides, which have extremely similar physical properties and might obstruct the real glucose concentration.

A few particular interesting sensor concepts are related to plasmonics-based systems. Plasmonics, or noble metal nanoparticle optics, utilize the large number of quasi-free conduction electrons in noble metals [8,9]. If a gold nanoparticle is illuminated by an external light field of appropriate wavelength, the quasi-free conduction electrons oscillate with respect to the fixed ionic background of the particle. As a consequence, this resonance is associated with so-called local near-fields in the close vicinity of the particle, which are significantly stronger than the far-field plane wave intensity. These local fields can be utilized for so-called refractive index sensors [10,11,12]. If an analyte molecular enters the near-fields, the far-field resonance will spectrally shift. This shift can be used as a highly sensitive sensor down to a few or even a single molecule [13,14]. However, without appropriate functionalization, such a sensor cannot distinguish different analyte molecules, as it only reports refractive index changes. While functionalization is possible, it is often highly complex, still sensitive to a certain cross-talk with similar molecules, and also tends to degrade rather quickly [15,16]. All of these points are, in particular, valid for complex and highly multi-entity environments, such as the human blood and any other body fluids.

Infrared spectroscopy, on the other hand, is known for its supreme specificity [17]. The infrared spectra of biomolecules show characteristic absorption features which allow to unambiguously identify molecular species. This spectral region is therefore often called the fingerprint region and the spectral signatures the molecular fingerprints. However, while specificity is excellent, there is little sensitivity. In general, large amounts of analyte are needed in order to acquire infrared absorption spectra. Here, again, the complex nature of the investigated samples comes as a challenge.

Fortunately, it has been shown that the best of both of these techniques can be combined in surface enhanced infrared absorption (SEIRA) spectroscopy [18]. SEIRA spectroscopy has been studied in great detail in order to uncover the basic working principle [19]. Generally speaking, the utilized plasmonic nanoparticles or nanoantennas are designed such that their plasmonic resonances are close to the molecular absorption bands. When the target molecular species enters the locally enhanced near-fields of the antennas, the vibrational modes and the plasmonic mode couple. As a consequence, the very strong and bright optical response of the plasmonic antenna is imprinted with the signature of the molecular species [19]. An enhancement of up to six orders of magnitude can be observed [18]. This allows to measure very small amounts of analyte in nanoscale volumes while simultaneously allowing to unambiguously identify the molecular species based on the unique vibrational signatures [20,21,22,23]. The basic working principle of SEIRA has been fully understood, making it a routine method for the investigation of other systems, species, or entities. Besides the observation of molecular species, for example during folding and unfolding of proteins [24,25], SEIRA has also been used for the investigation of the plasmonic resonances themselves, e.g., for the extend and intensity of the local near-fields [26,27].

## 2. Materials and Methods

**Figure 1:**
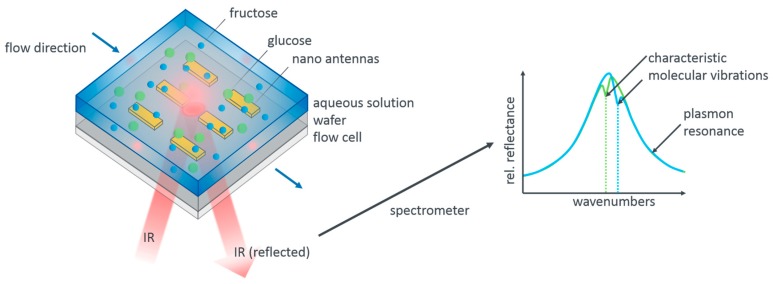
Measurement principle and sensor design. Aqueous pure glucose and fructose solutions as well as mixed solutions are flushed over plasmonic nanoatennas resonant with the characteristic mid-infrared vibrational modes of these monosaccharaides. The tailored flow cell allows to simultaneously acquire reflectance spectra, as sketched on the right hand side. The reflectance peaks of the antennas are modified by the vibrational signatures of the molecular species present in the analyte. From their spectral position the molecular species can be determined, from their modulation depth, in principle, the concentration, given appropriate evaluation and calibration, which is the key result reported here.

Glucose and fructose, as our model system, show, in fact, distinct vibrational modes in the infrared spectral range [28]. This fact allows us to distinguish these two species. Figure 1 illustrates our measurement concept. Resonant gold nanoantennas are incorporated into a flow cell which allows us to flush different aqueous analyte solutions over the antennas while simultaneously acquiring infrared reflection spectra. The reflection spectra contain the signature of the plasmonic resonance as well as the imprinted vibrational modes of the analyte molecules. As sketched, these differ for glucose and fructose. Apart from the mere presence of the analyte molecules, it should be possible to also extract the concentration of the molecules from the measurements as a higher concentration leads to a larger modulation depth of the vibrational features.

While the general concept is straightforward, there are in fact a number of challenges when aiming for a quantitative analysis. Firstly, the contribution of the plasmonic resonance has to be removed from the data. This is in general achieved by the so-called baseline correction. However, the exact modulation of the remaining features is found to depend significantly on the exact baseline. While this is of no further concern for identifying a molecular species, it is a significant hurdle in determining quantitative concentrations. We thus need a stable routine that allows us to “calibrate” our sensor scheme for concentration levels, that is, correlate the modulation depth of the molecular features with the concentration of molecules present. This calibration must not be disturbed by varying baseline corrections. We are going to show that we are indeed able, using a global baseline correction and an adaptive algorithm for basis function approximation, to extract these absolute values.

**Figure 2:**
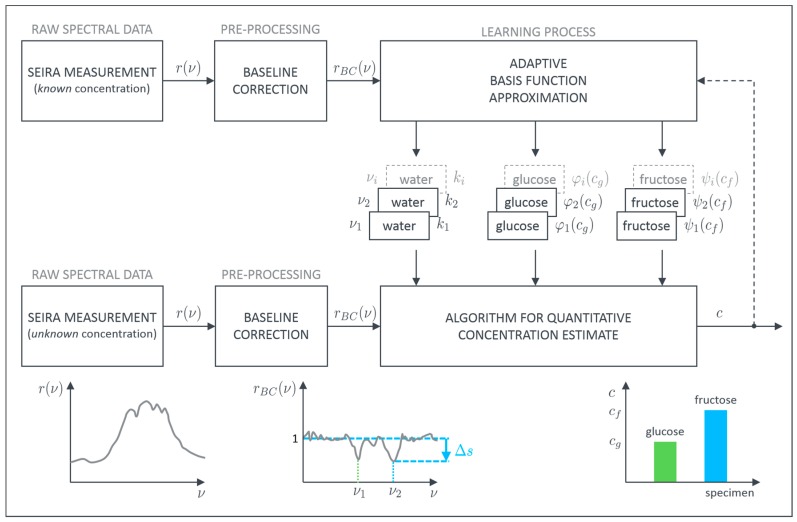
Schematic description of the developed adaptive process for evaluation of surface enhanced infrared absorption (SEIRA) measurement data to quantitatively estimate glucose and fructose concentrations in aqueous solutions.

Within the scope of this work, two different monosaccharides are examined with the proposed method. Due to their importance in health science, aqueous solutions of monosaccharaides are investigated. Glucose is a highly important biological molecule and its concentration in the blood or other body fluids needs to be determined with high accuracy. The primary hurdle lies in the presence of other biomolecules in, e.g., the blood or tear liquid. In particular, the other monosaccharaides cause problems in identifying the correct concentration [29]. As a model system, we therefore utilize aqueous solutions of different glucose and fructose concentrations in order to establish a robust routine for quantitative concentration determination. The proposed procedure to achieve this goal is schematically depicted in Figure 2. 

As mentioned above, our sensor data is obtained from SEIRA measurements. We collected a large set of reflective SEIRA spectra of different pure glucose and fructose as well as mixed solutions, as published elsewhere [28]. In brief: We utilize a reflection flow cell in inverse geometry, which is flushed via attached tube connectors that transport the desired solutions into and out of the flow cell. The key parts of our sensor are the different linear gold antenna arrays which were fabricated with electron beam lithography on top of IR transparent calcium fluoride substrates. The choice of the plasmonic element is motivated by the following aspects: Ease of structure fabrication, chemical stability, quality of the plasmonic resonance in terms of linewidth and amplitude, as well as resonance position. While there are many highly sophisticated structures and systems, which include, among the noble metals, also graphene [20,30,31,32,33,34], we choose the most straightforward realization via dipolar gold nanoantennas. These antennas have shown excellent quality factors, large dipole moments, are chemically inert, and can be easily fabricated via many bottom-up and top-down techniques. All of these aspects are also highly relevant for future applications. The geometrical parameters of the nanoantennas are chosen such as to exhibit a plasmon resonance at the spectral position of the targeted molecular vibrations of glucose and fructose, resulting in a length of 3500 nm, width, and thickness of 100 nm (over a 2 nm chromium adhesion layer), periodicity 4500 nm in x direction and 3000 nm in y direction [35,36]. For the spectral measurements, we use a commercial FTIR spectrometer (Bruker VERTEX 80, Bruker Optik GmbH, 76275 Ettlingen, Germany) and an optical microscope (Bruker Hyperion 2000, Schwarzschild objective with 15-fold magnification, NA = 0.4, Bruker Optik GmbH, 76275 Ettlingen, Germany). The spectra are measured with a nitrogen-cooled mercury cadmium telluride (MCT) detector and referenced to a gold mirror. The measurements spot is about 90 μm × 90 μm. Top-down electron beam lithography is a very well established technique leading to highly uniform structures which result in highly uniform optical properties of the elements. Additionally, the measured area contains a significant number of elements, leading to an effective ensemble averaging, minimizing the contribution of the individual, single object.

In general each SEIRA spectrum is a combination of the signature of the plasmonic resonance and the vibrational features which are characteristic for the molecular species. Since we are interested in the pure vibrational spectrum of the examined specimens, the plasmonic background has to be removed first. This can be achieved with, e.g., asymmetric least squares smoothing (ALSS) for baseline correction [37]. Thereby, it is possible to reconstruct the unperturbed plasmonic resonance or at least approximate it sufficiently. Afterwards, the pure vibrational data $r_{BC}$ is obtained by dividing the measured SEIRA spectrum by the reconstructed baseline. The so-called baseline corrected spectra, then, only contain the pure vibrational information showing up as peaks at their specific wavenumbers $\nu_{i}$. In principle, these peaks contain not only information about the presence of the respective molecule but also information about the absolute concentration. In order to extract this quantitative information, we developed an algorithm to evaluate the contribution of each specimen to the height of these characteristic peaks. The basic assumption for our algorithm is that a superposition principle can be applied. If this is valid, each specimen can be evaluated separately and the overall sum of all individual parts represents the measured height.

We expect the contribution of pure water to be constant ($k_{i}$), whereas the contribution of glucose ($\varphi_{i}$) or fructose ($\psi_{i}$) depends on the concentrations (c) of the dissolved monosaccharides. $\varphi_{i}$ and $\psi_{i}$ are the so-called basis functions which can be, in the most general form, arbitrarily shaped (e.g., polynomial, exponential, or other). To model the correlation between the level of monosaccharide-concentration and the resulting height of the peaks at the characteristic wavenumbers we can choose a suitable basis function for each specimen. This can be written as

(1) $$\Delta s_{g}\left(\nu_{i}\right)={\tilde{\varphi }}_{i}\left(c_{g}\right)-k_{i}=\varphi_{i}\left(c_{g}\right)$$

and

(2) $$\Delta s_{f}\left(\nu_{i}\right)={\tilde{\psi }}_{i}\left(c_{f}\right)-k_{i}=\psi_{i}\left(c_{f}\right),$$

resulting in

(3) $$\Delta s\left(\nu_{i}\right)=\Delta s_{g}\left(\nu_{i}\right)+\Delta s_{f}\left(\nu_{i}\right)=\varphi_{i}\left(c_{g}\right)+\psi_{i}\left(c_{f}\right),$$

where Δs is the height of the peak at the characteristic wavenumber. As we want to determine the absolute concentrations from our measurements, one has to solve a system of nonlinear equations for arbitrary basis functions $\varphi_{i}$ and $\psi_{i}$. Introducing $x={\left[c_{g}\;\;c_{f}\right]}^{T}$ and using (3) we obtain

(4) $$\underset{:=F\left(x\right)}{\underbrace{\left[\begin{array}{c} \varphi_{1}\left(x_{1}\right)+\psi_{1}\left(x_{2}\right)-\Delta s_{m}\left(\nu_{1}\right)\\ \vdots \\ \varphi_{n}\left(x_{1}\right)+\psi_{n}\left(x_{2}\right)-\Delta s_{m}\left(\nu_{n}\right) \end{array}\right]}}=0,\;n\geq 2$$

with $\Delta s_{m}$ being the measured peak heights and n being the number of characteristic wavenumbers which shall be evaluated. If n>2 holds, a least squares solution [38] is pursued given by

(5) $$x^{*}=\underset{\;}{\max}\left(\arg\underset{x}{\min}F{\left(x\right)}^{T}F\left(x\right),0\right).$$

If the calculations should yield negative concentrations, they are automatically set to zero, since they are from a physical point of view infeasible. 

The developed algorithm allows for a precise estimation of the concentrations for glucose and fructose. Importantly, the results of further measurements can be used as additional feedback to adapt the basis functions in order to improve the process quality and accuracy on the fly.

## 3. Results

Three different SEIRA measurement cycles are recorded to test and evaluate the proposed procedure. The pure glucose and fructose solutions are used to train our model, that is, determine the basis functions as well as the respective peak contributions to each of the characteristic spectral signatures as a function of the absolute concentration. Afterwards, we can analyze the mixed solutions which we assume as the solutions of “unknown” concentration in order to test the accuracy of our evaluation procedure.

**Figure 3:**
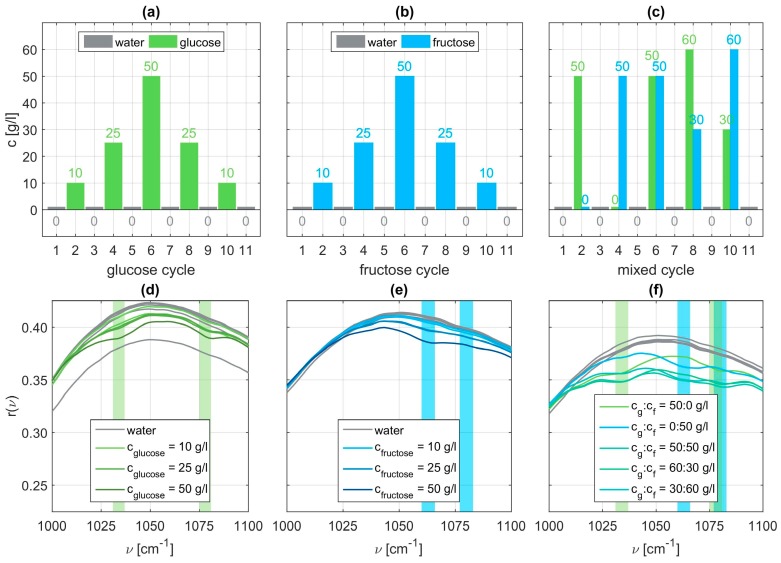
Employed measurement cycles with different concentrations of glucose (**a**), fructose (**b**), or both (**c**). Panels (**d**) to (**f**) show the corresponding relative reflectance of each of the eleven SEIRA measurement sets with the expected fingerprint region for molecular vibrations of glucose and fructose highlighted in light green or light blue, respectively.

**Figure A1:**
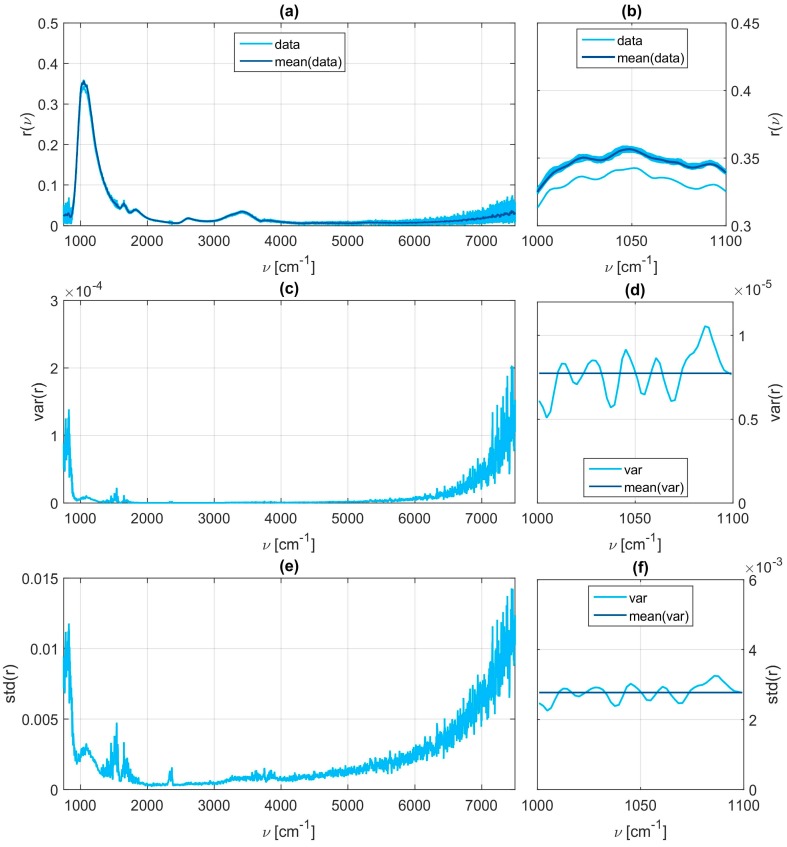
Statistical analysis of exemplary measurement data. The shown reflectance (**a**,**b**) results from a mixed solution with 50 g/l glucose and 50 g/l fructose. The measurement data consist of thirty individual spectra. The variance and the standard deviation between those spectra are calculated for each wavenumber and depicted in panels (**c**,**d**) and (**e**,**f**) respectively. The chosen measurement set reveals one of the largest variances because the reflectance of one spectra exhibits an almost constant offset. However, the qualitative features are preserved.

**Figure A2:**
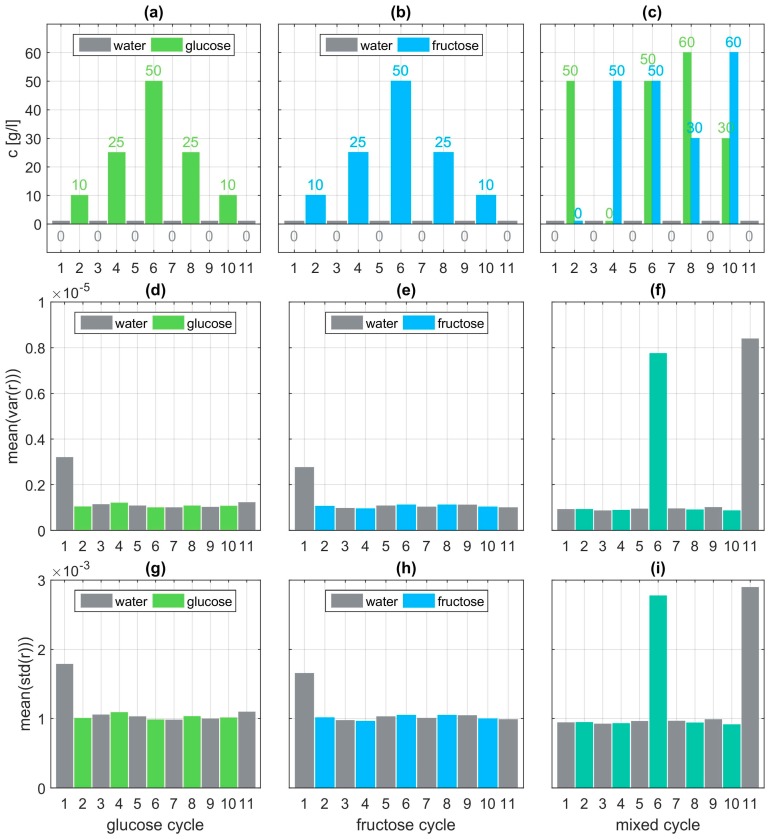
Statistical analysis of the measurement sets for each of the three cycles. The glucose/fructose concentration for each set is depicted in panels (**a**–**c**). The corresponding mean value of the variance within the fingerprint region ranging from 1000–1100 cm^−1^ (compare to panel (d) of Figure A1) is visualized in panels (**d**–**f**). The corresponding mean value of the standard deviation is depicted in panels (**g**–**i**).

Within each cycle, eleven sets of dilutions are examined. In the glucose cycle concentrations of 10, 25, and 50 g/l glucose are dissolved in water. Before, after, and between each aqueous solution, the flow cell of the sensor is flushed with pure water. On the one hand, this shall remove all residuals of the monosaccharide which could spoil subsequent measurements. On the other hand, a reference measurement for comparison to the aqueous solutions is recorded [28]. The order of execution is depicted in Figure 3 in panels (a) to (c). Within each of the eleven sets thirty individual spectra are taken. This not only allows for checking repeatability but also averaging over these thirty samples to give a more accurate and smoother representative measurement for further evaluation. A brief statistical analysis is provided in the Appendix A (Figure A1 and Figure A2). In a second measurement cycle the order of execution is repeated using fructose instead of glucose. During a last cycle mixed aqueous solutions containing both specimens with different varying concentrations are examined. All measurement data and all MATLAB related files for their evaluation are provided in the Supplementary Materials for the interested reader.

Exemplary SEIRA reflectance spectra for all three sets are shown in the Figure 3 panels (d) to (f). We can clearly identify vibrational signatures characteristic for glucose at $\nu =1034\;{\mathrm{cm}}^{-1}$ and $\nu =1078\;{\mathrm{cm}}^{-1}$(light green bars), whereas for fructose the vibrational signatures occur around $\nu =1063\;{\mathrm{cm}}^{-1}$ and $\nu =1080\;{\mathrm{cm}}^{-1}$ (light blue bars). The identified characteristic wavenumbers are in accordance with the ones reported in literature [39]. These vibrations can be identified as stretching vibrations of C-C and C-O bonds of the glucose ring or the fructose ring respectively. In case of the mixed solutions, we clearly observe the vibrational signatures of both molecular specimens. Examining the spectra closely, it is also obvious that the modulation depth is a function of the concentration. A higher respective concentration leads to a more pronounced vibrational feature. From these raw spectra it is also obvious that the mere presence of the molecular species can be easily and straightforwardly determined whereas the absolute concentration can only be estimated at best. This shortcoming we want to address next.

### 3.1. Pre-Processing the SEIRA Measurement

**Figure 4:**
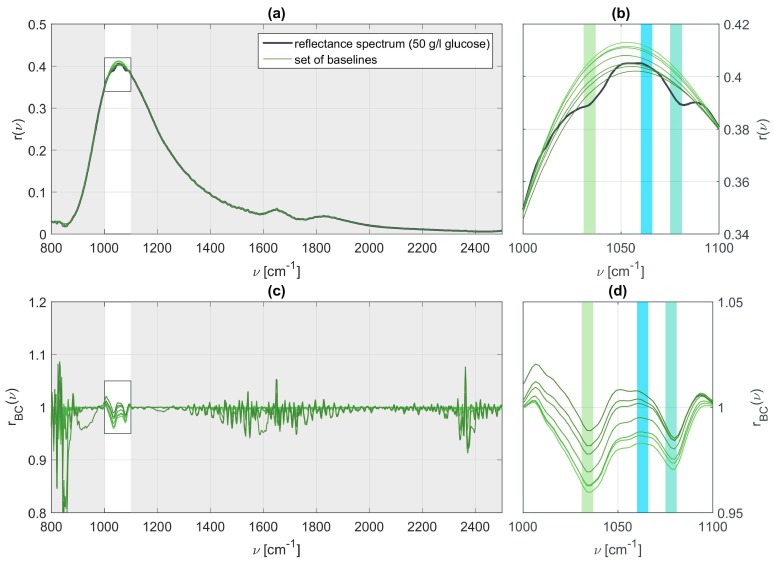
(**a**) Relative reflectance measured for 50 g/l aqueous glucose together with a set of baselines for varying parameters λ; (**b**) Zoom in on the relevant range of wavenumbers at which vibrational resonances are expected to appear for glucose and fructose; (**c**,**d**) Baseline corrected signals and the influence of λ on the vibrational features.

In order to extract the vibrational features of the present molecular specimen and determine its concentration we apply a slightly modified version of the ALSS algorithm [37] to the SEIRA data. The original version requires two weighting factors to be chosen. The first parameter λ determines how smoothly the reconstructed baseline is shaped. The second parameter p is responsible for the allowed asymmetry of the line shape. The algorithm is modified such that it allows to exclude parts of the fitting range within a certain neighborhood of the identified or known characteristic wavenumbers of the molecular species in the SEIRA measurement as the line is strongly deformed here. We performed a sensitivity analysis for the ALSS parameters which revealed that the absolute peak values of the vibration features in the baseline corrected spectra strongly depend on the chosen parameters. We have made different choices for the parameters λ and p, leading to different baselines. Panel (a) of Figure 4 shows a SEIRA measurement for a 50 g/l glucose solution and panel (b) a zoom into the spectral region of interest. The green curves in both panels represent different possible base lines. All of these baselines could be identified as reasonable but clearly different baselines, as is particularly obvious from the full reflectance spectrum and baselines depicted in panel (a). Panels (c) and (d) of Figure 4 display the baseline corrected SEIRA spectrum for the different baselines. The spectral position of the vibrational features is well retained but the absolute values, which are related to the actual concentration, are lost. For all the spectra it is possible to determine the presence of glucose but the concentration cannot be determined. These results clearly show that the choice of the baseline is of utmost importance and that the baseline must be chosen for all measurements simultaneously and consistently in order to retain quantitative information. Hence, the parameters are kept constant and set to λ=500, p=0.99, and $\nu_{excl.}\in \left[1000,\;1100\right]$ for comparability between all data sets.

**Figure 5:**
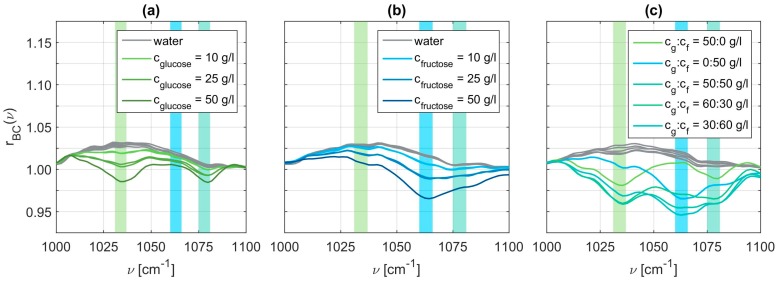
Relative reflectance of baseline corrected signals with λ=500 and p=0.99 for glucose cycle (**a**), fructose cycle (**b**), and mixed cycle (**c**).

Using these baseline correction values, we obtained the pure vibrational spectra. Figure 5 displays exemplary spectra for all three cases, that is, the pure glucose solutions in panel (a), the pure fructose solutions in panel (b), and the mixed solutions in panel (c). These spectra clearly exhibit an increasing modulation depth for increasing concentrations, as expected. The vibrational spectra of the mixed glucose and fructose solutions are rather complicated to interpret as they contain all the vibrational modes of glucose and fructose. The characteristic wavenumbers of glucose are determined to be at 1034 cm^−1^ and 1078 cm^−1^, whereas we obtain 1063 cm^−1^ and 1080 cm^−1^ for fructose. Since the second characteristic wavenumber of glucose and fructose are spectrally close together, we choose to only evaluate the peaks at $\nu ={\left[1034,\;1063,\;1078\right]}^{T}\;{\mathrm{cm}}^{-1}$ in the following.

The next question arising is related to the relative weight of each component in a certain peak, that is, the question whether or not each peak can be decomposed in its relative composition. Only if this is possible, the absolute concentrations, particularly in the mixed solutions, can be determined. For this purpose, we apply the proposed algorithm to the preprocessed data.

### 3.2. Basis Function Approximation

**Figure 6:**
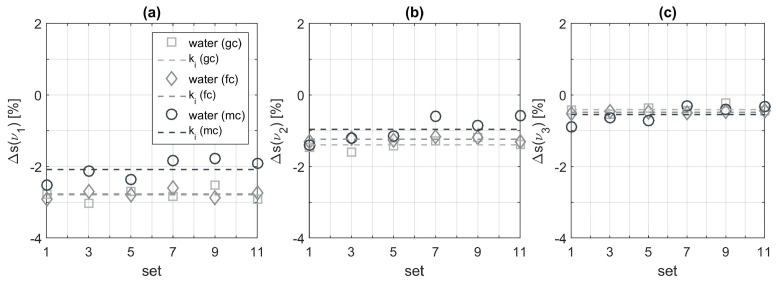
Signal peaks Δs evaluated at the three characteristic wavenumbers $\nu_{1}=1034\;{\mathrm{cm}}^{-1}$ (**a**), $\nu_{2}=1063\;{\mathrm{cm}}^{-1}$ (**b**), and $\nu_{3}=1078\;{\mathrm{cm}}^{-1}$ (**c**) originating from pure water for each of the three measurement cycles (gc = glucose cycle, fc = fructose cycle, and mc = mixed cycle).

When evaluating the extracted peaks of pure water measurements we obtain good agreement with the expected constant values for the glucose and fructose cycle which are depicted in panels (a) to (c) of Figure 6. The peaks obtained from the mixed cycle measurements deviate more around their average. A possible explanation could be that not all residues from previously measured aqueous solutions have been flushed out of the flow cell or are still sticking to the sensor surface. These deviations behave quite similar for all three examined wavenumbers. However, we also observe variations of the calculated mean values $k_{i}$ when comparing the different measurement cycles. In particular, this is visible at 1034 cm^−1^ in panel (a) and at 1063 cm^−1^ in panel (b). At this point we are not sure about the origin of these deviations. In order to include this influence, we decided to design the analysis routine adapted to the sensor data. This means that for each measurement cycle a calibration is carried out to identify the constants $k_{i}$ of Equations (1) and (2), characterizing the influence of pure water.

**Figure 7:**
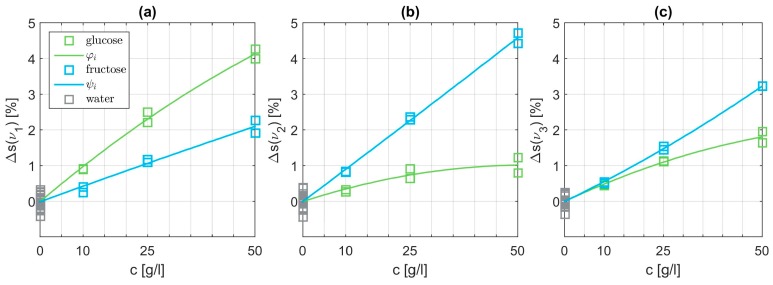
Signal peaks Δs evaluated at the three characteristic wavenumbers $\nu_{1}=1034\;{\mathrm{cm}}^{-1}$ (**a**), $\nu_{2}=1063\;{\mathrm{cm}}^{-1}$. (**b**), and $\nu_{3}=1078\;{\mathrm{cm}}^{-1}$ (**c**) originating from pure glucose or pure fructose, respectively. The content of pure water is already subtracted using the corresponding average value. The displayed basis functions $\varphi_{i}$ and $\psi_{i}$ are quadratic polynomials.

Subsequently, we analyze the SEIRA data for aqueous solutions containing only one monosaccharide. In a first step, we subtract the influence from pure water following Equations (1) and (2). Then, we have to choose suitable basis functions which are able to fit the data points best. Polynomials provided the optimum results. Starting from a simple linear function, the degree of the polynomials was tested up to an order of three. The polynomial basis functions $\varphi_{i}$ and $\psi_{i}$ are parametrized to match the data points best in a least squares sense. An exemplary combination of fitted basis functions is depicted in Figure 7. The shape differs clearly at each of the three characteristic wavenumbers. Panel (a) at $\nu_{1}=1034\;{\mathrm{cm}}^{-1}$ demonstrates the dominant influence of the glucose level as expected. However, interestingly, the influence of fructose is not negligible. At $\nu_{2}=1063\;{\mathrm{cm}}^{-1}$ it is just the opposite as displayed in panel (b). In the vicinity of $\nu_{3}=1078\;{\mathrm{cm}}^{-1}$ we expect resonances for both specimens visualized in panel (c). Overall, the basis functions of glucose and fructose can be matched quite well with quadratic polynomials.

### 3.3. Validation of Algorithm for Quantitative Concentration Estimate

**Figure 8:**
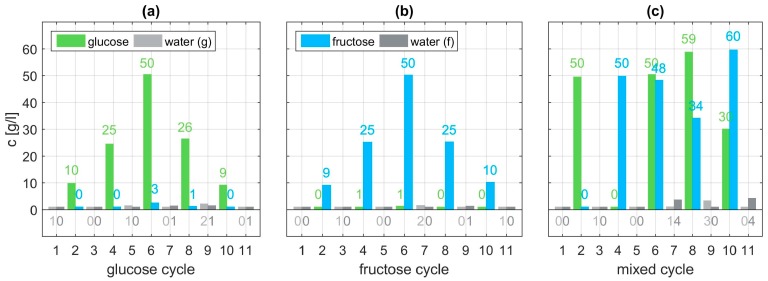
Estimated levels of concentration for the glucose cycle (**a**), fructose cycle (**b**), and mixed cycle (**c**) using cycle-specific constants $k_{i}$, quadratic polynomials $\varphi_{i}$ and linear basis functions $\psi_{i}$.

Having identified suitable basis functions $\varphi_{i}$ and $\psi_{i}$, the system of nonlinear Equation (4) can be solved in a least squares sense. For each of the thirty three measurement sets the concentrations are calculated using Equation (5) and compared to the expected value. For different combinations of polynomials as basis functions a comparison of the resulting RMS errors and mean errors are listed in Table 1. The best results are achieved with $\varphi_{i}$ and $\psi_{i}$ being quadratic polynomials with regards to the error mean value. The estimated concentrations are depicted in Figure 8 and exhibit very good agreement with the set values (Figure 3). Estimation of the concentrations of the glucose and fructose cycle, shown in panel (a) and (b), are expected to match well, since they have been used as training data for the algorithm. However, the algorithm also proves to be successful using the validation data (mixed cycle) depicted in panel (c). Overall, analyzing the concentration estimate for all three cycles a mean deviation of 0.71 g/l is achieved with the proposed procedure. With regards to the highest employed concentration of 60 g/l this mean error is below 1.2%.

## 4. Discussion

The results demonstrate that a concentration of monosaccharides such as glucose and fructose can be reliably and quantitatively sensed down to at least 10 g/l. The method not only works for aqueous solutions with pure glucose or pure fructose but also for mixed solutions containing both specimens. The non-invasive sensor principle relies on surface enhanced infrared absorption (SEIRA) spectroscopy of glucose and fructose in the mid-infrared spectral range. After preprocessing the SEIRA data with a baseline correction method, a new and adaptive algorithm is employed to reconstruct the concentration levels of glucose and fructose within aqueous solutions. Being able to choose arbitrarily shaped basis functions for any number of characteristic wavenumbers renders this algorithm highly flexible. The extension to incorporate additional monosaccharides is straightforward. Moreover, the structure of the algorithm facilitates its application to machine learning. Adapting or learning the basis functions and their parametrization could be achieved in an “online fashion”. This could be especially interesting for a sensor which senses glucose concentrations in the human ocular fluid or interstitial fluid that can be directly correlated to the blood glucose concentrations [7]. However, the measurement setup has to be improved in order to increase the sensitivity further to achieve sensitivity below 10 g/l, eventually down to physiological concentrations of mg/l. 

In order to explore the limitations of the proposed methods regarding robustness and generality further experiments are required. Other monosaccharides, as well as maltose as a biologically relevant double sugar, should be added as analytes, not only to identify their fingerprint resonances but also to validate whether the method is robust enough to accurately predict the concentrations of glucose and fructose even in more complex environments. Furthermore, measurements with aqueous solutions containing glucose or fructose with concentrations below 10 g/l have to be taken in future research.

## Figures and Tables

**Table 1:** Comparison of RMS errors, mean errors, and maximum errors for the estimated concentrations resulting from different combinations of polynomial basis functions $\varphi_{i}$ and $\psi_{i}$ with order $n_{glucose}$ or $n_{fructose}$ respectively.

$n_{glucose}$	$n_{fructose}$	$e_{rms}\;[g/l]$	$e_{mean}\;[g/l]$	$e_{max}\;[g/l]$
1	1	1.27	0.81	3.86
1	2	1.27	0.77	3.98
1	3	1.89	0.95	10.02
2	1	1.20	0.73	4.13
2	2	1.23	0.71	4.29
2	3	1.67	0.88	8.36
3	1	1.51	0.80	8.03
3	2	1.53	0.79	8.24
3	3	1.69	0.90	6.50

## Supplementary Materials

The following are available online at https://www.mdpi.com/1424-8220/19/14/3053/s1.

## Appendix A

A brief statistical analysis is done to proof the repeatability of the measurements. Within each measurement set thirty individual spectra are taken. For an exemplary set, namely the one with 50 g/l glucose and 50 g/l fructose dissolved in water, the individual spectra are depicted in panel (a) of Figure A1. Within the interested fingerprint region all spectra exhibit a very similar qualitative behavior (panel (b)). For the shown set, which is a worst case example, only one spectrum differs significantly from the others. Since we normalize the reflectance spectra with the baseline correction method afterwards in our data analysis procedure this offset becomes negligible anyways. Within the fingerprint region around 1000 to 1100 cm^−1^ the mean value of the variance (panel (d)) and the standard deviation (panel (f)) are below 10^−5^ and 4 × 10^−3^ respectively. Only for wavenumbers below 1000 cm^−1^ or above 6000 cm^−1^ the variance and standard deviation increase which is shown in panels (c) and (e). These inaccuracies in the border regions can be explained with the sensor design. It is tuned to provide best measurement results for the fingerprint region.

Repeating this statistical analysis for all measurement sets provides a base to judge the quality of the measurements. The results are depicted in Figure A2. In panels (a) to (c) the concentrations of glucose and fructose are visualized. The calculated mean values of the variances and the standard deviations within the fingerprint region are illustrated in panels (d) to (f) and panels (g) to (i) respectively. Only the first water measurement of the glucose and fructose cycle exhibit a slightly higher variance compared to the other sets of the corresponding cycle. Measurement sets six and eleven of the mixed cycle display the highest variance. For both sets, an individual spectrum had an almost constant offset in the reflectance as visualized in panel (b) of Figure A1. However, this offset becomes negligible due to the averaging over the thirty spectra and due to the normalization through the baseline correction method.
